# Gambling During the Covid-19 Pandemic

**DOI:** 10.1192/j.eurpsy.2022.352

**Published:** 2022-09-01

**Authors:** M. Skelin, A. Puljić

**Affiliations:** The University Psychiatric Hospital Vrapče, Department For Addictive Disorders, ZAGREB, Croatia

**Keywords:** gambling, sports betting, Covid-19

## Abstract

**Introduction:**

With the Covid-19 pandemic numerous questions about the behaviour of gambling addicts have risen, with the lockdown causing a lack of structure, peer supervision and support. The first reports have suggested an increase in activity and riskier choices.

**Objectives:**

Our aim was to explore how the Covid-19 pandemic has influenced gambling habits.

**Methods:**

Data was collected from companies in Germany and Croatia which provide online gambling services, and statistically analyzed.

**Results:**

In Germany in April 2020, there was a 51.19% decrease in number of players when compared to April 2019, but a 116.46% rise in the number of tickets per player. In comparison, in April 2021 a rise of 704.43% occurred in number of active players compared to April 2020, with a 277.56% increase in ballots and a decrease in number of tickets per player by 53%. Additional results showed a 1.2% decrease in spendings on sports events in April 2020 compared to April 2019, but a 277.88% increase for sporting events spendings in April 2021. Preliminary results from Croatia show an increase in online gambling activities.

**Conclusions:**

In 2020, despite a drop in active players, the increase in stakes and frequency of play resulted only in a slight decrease in sporting events spendings. In 2021 the recorded increase in all categories except in frequency, points to the idea that restoring life to usual rhythm reduces the frequency of an individual’s play. In conclusion, further research and monitoring of gambling addicts’ behaviour in the “new normal” is needed.

**Disclosure:**

No significant relationships.

